# Evaluating commercial multimodal AI for diabetic eye screening and implications for an alternative regulatory pathway

**DOI:** 10.1038/s41746-025-02216-7

**Published:** 2025-12-15

**Authors:** Matthew S. Hunt, Tinglong Dai, Michael D. Abràmoff

**Affiliations:** 1 Department of Ophthalmology and Visual Sciences. University of Iowa, Iowa City, IA USA; 2 Carey Business School, Johns Hopkins University, Baltimore, MD USA; 3 Hopkins Business of Health Initiative, Johns Hopkins University, Washington, DC, USA; 4 School of Nursing, Johns Hopkins University, Baltimore, MD USA; 5 Department of Electrical and Computer Engineering, University of Iowa, Iowa City, IA USA; 6 Veterans Affairs Medical Center, Iowa City, IA USA

**Keywords:** Retinal diseases, Health care economics, Health policy

## Abstract

Autonomous AI for diabetic eye examination is among the most validated and trusted medical AI systems, supported by extensive real-world evidence demonstrating safety, efficacy, improved outcomes, increased productivity, and cost savings. Yet its adoption remains limited. In contrast, commercially available off-the-shelf generative AI models (OTSAIs) are being rapidly tested in medical settings despite a lack of such real-world validation. These models have shown strong performance on medical reasoning tasks, prompting interest in their potential for clinical deployment. We evaluated four OTSAIs—GPT-4o and GPT-4o-mini (OpenAI, San Francisco, CA), Grok (xAI, San Francisco, CA), and Gemini (Google, Mountain View, CA)—on a specific diagnostic task: diabetic eye examination. The OTSAIs were *bundled* to ensure consistency, and performance was assessed using a level 3 reference standard, the publicly available Messidor-2 dataset. GPT-4o achieved the highest area under the receiver operator characteristic curve (AUC), 0.83. Grok achieved 0.63, and AUC was not calculable for Gemini. The AUC of retina specialists on the same task was estimated at 0.94, so the emergent performance of OTSAIs does not match that of clinical experts, nor does it approach FDA endpoints for consideration as a medical device. Nevertheless, as the performance of these OTSAIs approaches theoretical limits in the future, there might be a regulatory path through task-specific licensing by State Medical Boards for specific clinical tasks. This path may be modeled after licensing for physician assistants, where trust in the bundled OTSAI, to be used in an assistive fashion, is achieved through rigorous validation for safety and efficacy according to widely accepted regulatory considerations for both patient-facing AI, as well as for SaMD processes.

## Introduction

Autonomous artificial intelligence (AI) for the diabetic eye examination is one of the most validated and widely adopted medical AI use cases in the United States^1^. Real-world deployments, supported by randomized controlled trials and observational studies, have demonstrated improved patient outcomes^2,3^, expanded access to care, reduced health disparities^4^, and increased clinician productivity^5^ at lower cost^6,7^. Notably, this specific autonomous AI system also cleared critical ethical, regulatory, reimbursement, and workflow hurdles—establishing a viable path for future medical AI deployment^7–9^. Given these successes, one might expect other AI systems to quickly follow. Yet this has not occurred. Even the most widely used medical AI devices today reach fewer than 1% of the patients who could benefit^10^.

Trust in such AI requires rigorous validation along with substantial effort and risk to design, build, and validate autonomous AI models in alignment with the U.S. Food and Drug Administration’s (FDA) regulatory total product lifecycle (TPLC) framework for SaMD. Ensuring trust requires demonstrating high diagnostic accuracy^9^, mitigating bias^11^, and ensuring clinical interpretability^8^. Although ophthalmic foundation models offer promise for more rapid iteration through (implicit) priors^12^, none have yet advanced to FDA-cleared medical devices. At the same time, general-purpose, unsupervised (with respect to medical tasks), off-the-shelf multimodal AI models (OTSAIs)—such as ChatGPT (OpenAI, San Francisco, CA), Gemini (Google, Mountain View, CA), and Grok (xAI, San Francisco, CA)—have advanced rapidly in their capabilities. These OTSAIs are not trained for any specific medical task, nor are they marketed as medical devices. Thus, they differ fundamentally from purpose-built clinical models. They have demonstrated strong performance on standardized medical reasoning exams despite lacking explicit clinical training, including the Medical College Admission Test (MCAT), the United States Medical Licensing Examination (USMLE), and the Ophthalmic Knowledge Assessment Program (OKAP) Board preparation examination^13–15^. Early investigation has also looked at accuracy on medical imaging tasks^16^. However, none of these models has been evaluated as would be required if it were a medical device—such as through Good Clinical Practice (GCP)-compliant clinical trials using a level 1 reference standard within a real-world workflow, along with the many other requirements that have become standard for autonomous diagnostic AI devices^8^.

Because OTSAIs are not designed or trained for specific diagnostic tasks, their performance on such tasks is an *emergent* behavior, i.e., task performance is not a result of deliberate training or model optimization^17^. Although these unsupervised foundation models have likely ingested biomedical content during training, they were not deliberately trained or optimized for the diabetic retinopathy task. Thus, any above-chance performance on this task can be viewed as an emergent behavior rather than the result of task-specific training. In this way, OTSAIs are similar to physicians and other clinicians, who are regulated through a licensing process by State Medical Boards, in the US and many other jurisdictions. Potentially, OTSAIs might qualify for a similar licensing process, coupled with validation for specific tasks to assist clinicians.

However, to our knowledge, no OTSAI has been evaluated on a clinical task using performance standards consistent with established regulatory expectations for medical devices. Such an evaluation requires a clinical domain with well-defined, stakeholder-supported benchmarks—including alignment with developed FDA standards, established workflows, interpretable reference standards, and publicly available datasets. Ophthalmology, specifically the autonomous AI diabetic eye examination, offers a uniquely mature setting for this kind of assessment, with broadly accepted and validated level 3 reference datasets such as ROC and the publicly available Messidor-2 dataset, in combination with a widely supported and documented regulatory process^8,18–20^.

In this study, we evaluate the diagnostic performance of four OTSAIs to assess whether such OTSAIs could now, or perhaps in the future, meet the regulatory performance thresholds required of autonomous diagnostic devices aligned with current FDA standards.

## Results

### Reportability across models and prompting strategies

All containerized OpenAI and xAI models—GPT-4o, GPT-4o-mini, and Grok-2-vision—successfully returned valid, structured outputs in 100% of cases across the minimal, background, and few-shot prompting strategies. In contrast, Gemini-1.5-pro exhibited lower reportability, returning analyzable outputs in 85% of cases with minimal prompting, 89% with background prompting, and 100% with few-shot prompting.

### Receiver operating characteristic (ROC) analysis against a level 3 reference standard

AUC values for all models and prompting strategies are presented in Table 1.

**Table 1:** Sensitivity/specificity and (AUC) metrics for all combinations of model/prompting strategy for the models permitting ROC curve generation and set-point adjustment

Prompt	gpt-4o-2024-08-06	gpt-4o-mini	grok-2-vision-1212
Minimal	0.86/0.59 (0.83)	0.86/0.29 (0.66)	0.86/0.23 (0.63)
Background	0.88/0.63 (0.83)	0.86/0.29 (0.65)	0.86/0.23 (0.63)
Few-shot	0.86/0.56 (0.80)	0.85/0.21 (0.63)	0.86/0.25 (0.66)

Sensitivity/specificity pairs are at the set point for which the 95% CI lower bound is 80% sensitivity. Corresponding ROC curves and 95% CI are shown in Supplementary Fig. 2.

**Fig. 1:**
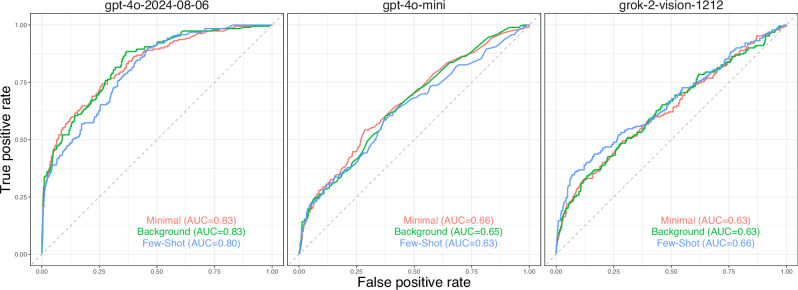
ROC Curves of each model under each prompting strategy. Each panel shows ROC curves for a given model. The curves are color-coded according to prompt strategy use. AUROC is shown for each model-strategy pair in the legend. The model “gpt-4o-2024-08-06” had the highest AUROC across all prompting strategies.

**Fig. 2:**
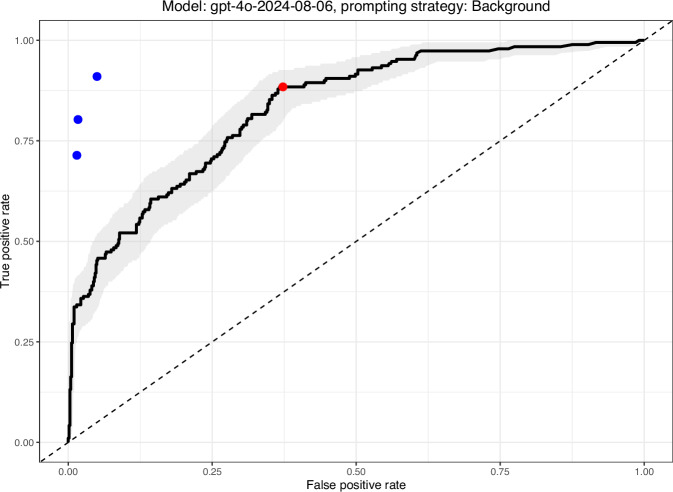
ROC curve with 95% confidence bounds for “gpt-4o-2024-08-06” using the “Background” prompting strategy. 95% confidence bounds were derived via bootstrapping (2000 stratified samples) using the pROC package in R. The red point marks the model set point at which the lower bound of the 95% confidence interval for sensitivity was at least 80%. The three blue points represent the sensitivity and specificity values for the three expert human graders.

GPT-4o-2024-08-06 achieved the highest AUC overall at 0.83. ROC curves for each model under each prompting strategy are shown in Fig. 1. A representative ROC curve for GPT-4o using background prompting, along with 95% confidence intervals, is shown in Fig. 2, with the selected operating point indicated in red. Sensitivity–specificity pairs for three human expert graders are shown for comparison; the estimated retina specialist AUC on the same dataset was 0.942^21^.

### Diagnostic performance compared to the level 3 reference standard

Sensitivity and specificity for each model and prompting strategy are shown in Table 1. GPT-4o-2024-08-06 demonstrated the highest overall performance across models, with comparable results using minimal and background prompting strategies. Across all prompting strategies, this model achieved a maximum sensitivity of 88% and specificity of 63% when using background prompting, as determined at the threshold where the lower bound of the 95% confidence interval for sensitivity exceeded 80%. Sensitivity and specificity for each model and prompting strategy without the set point is shown in Table 2.

**Table 2:** Sensitivity and specificity pairs for each model under each prompting strategy

Prompt	gpt-4o-2024-08-06	gpt-4o-mini	grok-2-vision-1212	gemini-1.5-pro
Minimal	0.54/0.91	0.10/0.99	0.24/0.94	0.45/0.84
Background	0.57/0.87	0.09/0.99	0.22/0.94	0.62/0.70
Few-shot	0.63/0.75	0.25/0.91	0.33/0.94	0.56/0.74

### False negative patterns and consistency across models

**Fig. 3:**
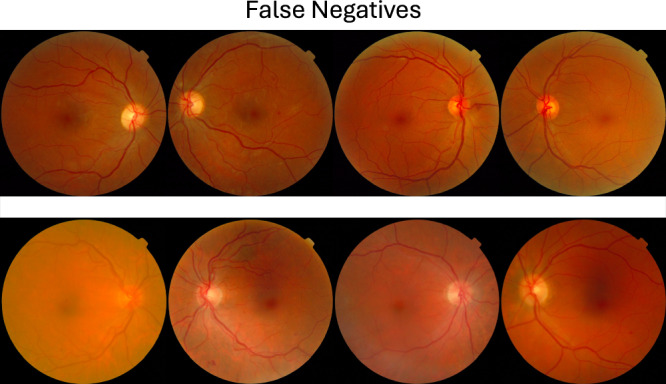
False negative predictions for “gpt-4o-2024-08-06” at the selected set-point for the “Background” prompting strategy. The false negative patients tended to have low-severity mild NPDR (ICDR score ≤2) and had dot-blot hemorrhages, qualifying them as mtmDR.

Analysis of false-negative predictions revealed limited overlap across models. Intersection over union (IoU) values for false-negative sets across prompting strategies were 0.35 for GPT-4o-2024-08-06, 0.24 for Grok-2-vision-1212, and 0.19 for GPT-4o-mini, indicating that GPT-4o-2024-08-06 produced the most consistent results across different prompting strategies. Representative false-negative cases for this model are shown in Fig. 3. All false-negative cases are shown in Supplementary Fig. 3.

## Discussion

In this study, we found that an off-the-shelf AI model (OTSAI), GPT-4o—despite not being explicitly trained for any medical imaging task—achieved an emergent AUC of 0.83, and a sensitivity of 88% and specificity of 63%, at the threshold meeting a lower bound of 80% sensitivity, against a level 3 reference standard, in detecting more-than-mild diabetic retinopathy. For context, retina specialists achieve an AUC of 0.94 on this same level 3 reference standard, Messidor-2^21^. Thus, OTSAIs still substantially underperform clinical experts. Given that performance against a level 3 reference standard typically overestimates performance against a level 1 standard—as required by the first autonomous-AI pivotal trial—by 10–30%^9,11^, these OTSAIs also would not meet regulatory thresholds for safety and efficacy as autonomous AI devices. Under current regulatory requirements, autonomous diagnostic AI systems are expected to exceed the lower bound of 80% for both sensitivity and specificity against a level 1 reference standard. It should be noted that the Messidor-2 dataset is publicly available and that it is possible this data was accessed when building the OTSAI. Consequently, our reported AUC, sensitivity, and specificity metrics may be taken as potentially inflated, upper bounds on accuracy.

In addition to substantially higher accuracy against a level 1 reference standard, OTSAIs would also require evaluation for demographic and clinical bias under GCP, adherence to ISO 13485 quality management system (QMS) and IEC 62304 software lifecycle standards, full alignment with the FDA’s TPLC, and many other regulatory guidelines for software as a medical device (SaMD)^22^. Table 3 lists requirements for an ISO 13485 compliant QMS; this would be a baseline set of standards to claim conformance for an OTSAI bundle for diabetic eye exams.

**Table 3:** Common standards for SaMD manufacturer adherence in the US^35^

Standard	Description
21 CFR Part 11	Electronic records; electronic signatures
21 CFR Part 50	Protection of human subjects
21 CFR Part 54	Financial disclosure by clinical investigators
21 CFR Part 56	Institutional review boards
21 CFR Part 803	Medical device reporting
21 CFR Part 806	Medical devices; reports of corrections and removals
21 CFR Part 807	Establishment registration and device listing for manufacturers
21 CFR Part 812	Investigational device exemptions
21 CFR Part 820	Quality system regulation
21 CFR Part 830	Unique device identification
ISO 13485	Medical devices—QMS—Requirements for regulatory purposes
IEC 62304	Medical device software—Software life cycle processes
ISO 14971	Medical devices – Application of risk management to medical devices
IEC 62366	Application of usability engineering to medical devices
ISO 14155	Clinical investigation of medical devices for human subjects—GCP
AAMI SW96	Standard for medical device security—security risk management for device manufacturers
ISO 15223	Symbols to be used with information to be supplied by the manufacturer
ISO 20417	Medical devices—information to be supplied by the manufacturer

Analysis of false-negative cases shows these most often occurred in less-severe cases—such as those with single retinal hemorrhages—which are also challenging for human experts (Fig. 3). This pattern may indicate that the model’s perceptual strategy approximates that of human experts, though further analysis into underlying mechanisms is beyond the scope of this pilot study.

This study has several limitations. First, the evaluation was conducted using a publicly available, retrospective dataset (Messidor-2) and a level 3 reference standard. While this standard is often used for benchmarking, it is not a level 1 prognostic standard, such as has been required for regulatory approval of autonomous AI. Such a validation would require a prospective study in a primary care setting with a level 1 reference standard (i.e., using a prognostic reference standard). Thus, the measured level 3 accuracy likely overestimates true performance. Given that multiple level 3 and 4 Messidor-2 standards are publicly available, including through the Kaggle competition website^23^, and likely the models ingested such data, this may have led to additional overestimation of accuracy. Optimal unbiased performance estimation would require validation on prospectively collected, preregistered, independent, access-controlled datasets—shielded from public exposure—corresponding to level 1–4 reference standards^8^. This is essential to remove any risk of training data leakage. However, conducting these studies demands substantial resources—often tens of millions of dollars—typically available only to private companies rather than academic research groups. Nonetheless, our open-source codebase and containerized pipeline are designed to support future external validations, which we identify as a priority for subsequent research.

Finally, this study could not evaluate performance in the clinical workflow, which may include operators with lower expertise—as was the case in the first pivotal trial—also likely leading to overestimation. It is important to note that we did not fine-tune or otherwise adapt these models for the task; exploring supervised optimization strategies (and their impact on both diagnostic accuracy and general medical reasoning performance) is beyond the scope of this pilot study. While these unsupervised models were not explicitly trained for our task, we recognize that their training on vast data likely endows them with some relevant knowledge—so their success is “emergent” only in the sense that no task-specific tuning was done. Only performance on retinal images was evaluated, and our findings may not generalize to other clinical domains or imaging modalities. However, autonomous AI for the diabetic eye exam has been a pioneering precedent in real-world implementation of AI, including AI ethics^4^, liability^24^, regulation^9^, RCTs^3^, and reimbursement^7^, and thus the policy and deployment considerations may extrapolate more broadly to medical AI. Future work should indeed examine these general-purpose models in additional clinical contexts. Finally, none of the many other requirements for autonomous AI as a medical device were evaluated, including alignment with an ISO 13485 compliant QMS, and the many other requirements for SaMD and AI explained above.

While the OTSAIs do not currently meet the criteria to qualify as regulated medical AI devices, their performance may improve over time and approach its theoretical limit—that of publicly available ophthalmologist performance on such narrowly defined diagnostic tasks. Although not currently clinically viable, OTSAIs already achieve expert-level performance on standardized medical reasoning assessments, including the MCAT, USMLE, and OKAP^13–15^, as well as softer medical competencies such as effective communication, ethics, and empathy^13,25^.

Looking ahead, as OTSAIs improve, one could envision an alternative regulatory path, through task-specific licensing and monitoring by organizations currently responsible for human expert licensing, such as the Federation of State Medical Boards. Medical professionals are typically regulated through state-based licensure systems, where they are evaluated on competencies including medical school graduation, residency completion, and performance on the medical-reasoning-based examinations (on which OTSAIs also do well), as well as continuing medical education (CME), to ensure that clinicians remain up to date with current standards of care. Importantly, licensed clinicians’ accuracy on specific clinical tasks is considered *emergent behavior*—i.e., arising from complex adaptive systems without either training nor accuracy assessment for specific diagnostic or therapeutic tasks^17^. In fact, only under very specific circumstances are physicians and other providers evaluated for diagnostic performance: in scientific studies designed for that purpose, or in malpractice investigations. Prior studies have shown that ophthalmologists’ sensitivity in the diabetic eye exam against level 1 prognostic reference standards range from 30% to 40%^26,27^, and would thus fail evaluation as a “medical device” under FDA criteria for an autonomous AI system. Under this pathway, OTSAIs would be *bundled* for a specific clinical task. Bundling would involve scoping and standardization of inputs (prompts and patient data, such as images) and outputs, setting of consistent thresholds, and validation of its performance on general medical tasks, including clinical knowledge and medical reasoning, as set forward previously. But, unlike human clinicians, whose accuracy on diagnostic tasks is assumed to be emergent, licensing for an OTSAI bundle for the diabetic eye exam would require explicit rigorous validation for that specific task to ensure trust in the bundled OTSAI. Such a pathway might parallel the licensing pathway for physician assistants, who are licensed to assist a licensed physician. It is generally accepted that the liability for the performance of medical device based autonomous AI falls on the AI creator^24,28,29^, and similarly we expect this would be the case for non-device, licensed bundled OTSAIs.

As set forward in the introduction, despite growing evidence—including from randomized controlled trials—that AI systems improve clinical outcomes^2,3^, increase access, reduce disparities^4^, enhance clinical productivity^5^, and lower costs^7^, the real-world adoption of AI in healthcare remains limited. As of 2025, more than 1000 AI-enabled medical devices have received FDA clearance, yet only a small subset have achieved meaningful uptake in clinical practice^1,30^. Regulatory complexity, uncertainty, and risk remain key barriers to broader adoption. The alternative licensing pathway could accelerate scalable deployment of bundled OTSAIs for such narrowly defined clinical tasks.

On the other hand, this pathway may not align with the overarching goal of optimizing patient outcomes. Current bioethical frameworks, which emphasize the dyadic physician–patient relationship, rather than centering on patient outcomes, do not adequately capture the broader tradeoffs involved in scaling high-performing autonomous AI^31^. Holding bundled OTSAIs only to the standard of mimicking average clinician performance—rather than surpassing it, as is the case for autonomous AI medical devices—may limit outcome improvement and essentially provide affordable substitution for clinicians, rather than more affordable care with superhuman accuracy. Autonomous AI devices that demonstrably outperform clinicians on objective clinical tasks may offer greater individual and societal benefit.

We have demonstrated that OTSAIs—despite not being explicitly trained for diagnostic tasks—can achieve reasonable accuracy on the diabetic eye exam, but still substantially underperform clinical experts, and do not meet regulatory standards for FDA-regulated autonomous AI medical devices. As one of the first works to formally evaluate such models, documenting such a “negative” finding, that OTSAIs are not on par with supervised AI, is scientifically meaningful by setting a benchmark clarifying the remaining performance gap. Further, this work provides a regulatory aligned, reproducible evaluation framework for OTSAIs. The publicly available code, including transparent prompt engineering, will facilitate replications of and expansions on our work. Multiple aspects set an example for alignment with regulatory expectations: (i) the use of prespecified operating point selection for sensitivity and specificity metrics; (ii) full adherence to TRIPOD LLM reporting standards; and (iii) explicit mapping of observed performance to FDA’s TPLC and ISO13485 quality management requirements. This study not only provides a confirmation of OTSAI underperformance relative to dedicated, supervised AI but offers a template for future evaluations of general-purpose models across clinical tasks.

While we introduce the concept of licensing OTSAIs, we acknowledge this is untested and that numerous legal and ethical questions remain, including those surrounding liability, bias, and patient benefit. Additionally, it is clear OTSAIs are not currently viable for clinical deployment. This discussion is a speculative and forward-looking conclusion and is intended to prompt proactive consideration of governance, rather than to propose an immediate policy change based on current evidence. Ultimately, any model implemented must be held to standards to avoid unacceptable risk, and outcomes improvement should remain the ultimate yardstick. We do not endorse this licensing approach outright but suggest it as one scenario to consider in view of the pressing need to expand AI access responsibly. Indeed, all stakeholders’ primary goal should be to improve patient outcomes, and delaying access to demonstrably beneficial AI represents a missed opportunity for improving outcomes at scale.

Looking ahead, as OTSAIs increasingly exhibit emergent clinical competencies, an alternative pathway may be needed to balance trust and scalability. A licensing-based framework—modeled after physician assistant licensing by Medical Boards—for bundled OTSAIs performing specific clinical tasks might accelerate deployment and scale the impact of beneficial AI, ultimately improving patient outcomes. Whether pursued through traditional medical device regulation or a licensing model for bundled OTSAIs, either pathway will require evidence of safety and security, in accordance with current regulatory considerations and guidelines. Ensuring trust, while maintaining transparency and accountability, will be essential to differentiate beneficial AI from those that pose unacceptable risk, all in pursuit of the shared goal of massively improving patient outcomes with trusted AI.

## Methods

### Dataset

For model evaluation, we used the publicly available Messidor-2 dataset, (https://www.adcis.net/en/third-party/messidor2/) which consists of 1748 de-identified macula-centered fundus images from 874 examinations of patients with diabetes. This dataset is available for public, noncommercial use and has previously been described in detail^21^. The images were acquired with a Topcon TRC NW6 nonmydriatic fundus camera (Topcon USA, Inc). A level 3 reference standard was provided by three clinicians and is also publicly available, defined as the presence or absence of more-than-mild diabetic retinopathy (mtmDR), currently operationalized as ETDRS grading of ≥35 and/or having (clinically significant) macular edema^32,33^, as per ETDRS. Any ETDRS grading ≥35 implies an International Clinical Diabetic Retinopathy (ICDR) severity scale score ≥2, indicating moderate or severe non-proliferative diabetic retinopathy (NPDR) or proliferative diabetic retinopathy^34^. Ground truth mtmDR labels were obtained using voting from the blinded grading of each image in the dataset by three world-renowned, expert retina specialists^21^. The number of patients in the dataset with mtmDR was 190, and the number of patients without mtmDR was 684, corresponding to a prevalence of 21.7%. This study used only publicly available, fully de-identified data (Messidor-2) and therefore did not require institutional review board approval or informed consent.

Each image in the dataset was preprocessed by cropping to the size of the circular retinal image. All images were downsampled to a size of 768 × 768 pixels for consistency of resolution across all models, as models from OpenAI and xAI automatically perform this downsampling prior to image processing.

### Models and prompt design

This study retrospectively evaluated existing, publicly available models. Specific, off-the-shelf, commercially available OTSAIs were selected for analysis. The models were “gpt-4o-2024-08-06” and “gpt-4o-mini” from OpenAI, “grok-2-vision-1212” from xAI, and “gemini-1.5-pro” from Google. All models were accessed via the application programming interfaces (APIs) provided using Python version 3.13.0, and all model responses were generated between February 13 and February 20, 2025. For all models, the “temperature” parameter was set to zero to minimize output randomness and promote selection of the most likely token sequence.

### Containerization

Each model was containerized as follows: model versions frozen on specific dates were accessed via application programming interfaces, with standardized structured prompts preceding image presentation for grading. Models were given standard instructions to produce responses in JavaScript Object Notation (JSON) format, which was subsequently programmatically post-processed. Three prompting strategies were evaluated across all models, as detailed in Supplementary Fig. 1:

- Minimal: the model was given basic system instructions and a request to return a Boolean grade for mtmDR in structured JSON.
- Background: the model was additionally supplied with background on ETDRS scoring definitions for diabetic retinopathy severity.
- Few-shot: each model was shown every image in our dataset, preceded by question–answer pairs consisting of five images with their ground-truth answers as contextual lead-up to the image being graded.

The desired structured JSON output was a dichotomous true or false value that was compared to the reference standard. Models were prompted to provide a mtmDR grade for every image (no ‘ungradable’ category). Reportability (promptability) was the percentage of queries that resulted in a properly JSON-formatted output. Patients for whom the mtmDR was not returned in properly structured JSON format for each of the two images were not analyzed. Only the properly reported outputs were included in the analyzed data. Sensitivity and specificity were calculated at the subject level (either eye having mtmDR).

### ROC analysis

ROC analysis was conducted at the subject level. For models that permitted probability extraction, we estimated confidence by summing likelihood of all tokens corresponding to a “true” Boolean value. These probabilities were then used to generate ROC curves.

For each model, we selected the operating point where the lower bound of the 95% confidence interval for sensitivity was ≥80% and computed the corresponding specificity. Confidence intervals bounds were derived via bootstrapping (2000 stratified samples) using the pROC package. Sensitivity–specificity pairs were also calculated for each of the three human graders by comparing each grader’s annotations against those of the other two, as previously described in ref. ^20^. An estimated AUC presenting expert human performance was computed based on the area enclosed by these three grader-derived points.

### False negative analysis

We identified all false negative cases for the best-performing OTSAI. Among subjects with false negative predictions, we computed the IoU for each model across all prompting strategies to assess consistency in error patterns.

## Supplementary information

OSTAI_supplementals_7_27_25

Tripod_LLM_Checklist

## Data Availability

The Python, Bash, and R code for this project is open-sourced and can be found at https://github.com/atthewmay/OTSAI_for_mtmDR_screening. The images and reference standard can be downloaded at https://medicine.uiowa.edu/eye/abramoff.
