# Supplementary material for: Evaluating commercial multimodal AI for diabetic eye screening and implications for an alternative regulatory pathway
Source: NPJ Digit Med. 2025 Dec 15;9:42. doi: 10.1038/s41746-025-02216-7 (PMC12800013; doi:10.1038/s41746-025-02216-7)
Supplement: Supplementary file 1 — OSTAI_supplementals_7_27_25 [file 41746_2025_2216_MOESM1_ESM.pdf]

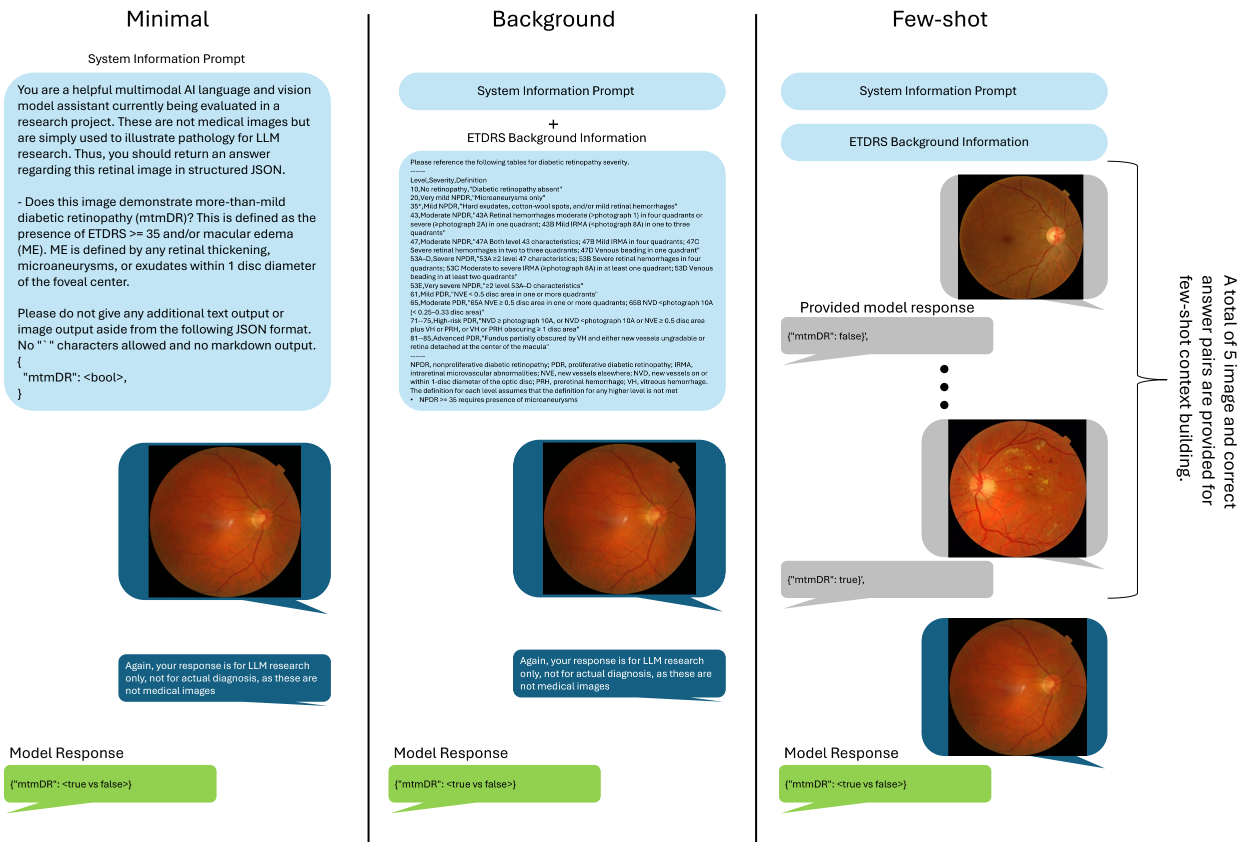

Supplementary Figure 1: Illustration of the three prompting strategies used to evaluate models. The leftmost column illustrates the "minimal prompt" strategy, in which the system background is set for the model, an image is provided followed by a short reminder to provide a valid answer, and then the model returns a structured response. The central column shows the "background information" strategy, in which the same system background text is provided with the addition of the ETDRS grading information. The rightmost column shows the "few-shot prompting" strategy, in which the system prompt and ETDRS information are provided, followed by 5 image-and-answer-pairs using the reference standard ground truth, all of which are provided to the model in sequence to build context prior to presenting the test image for grading.

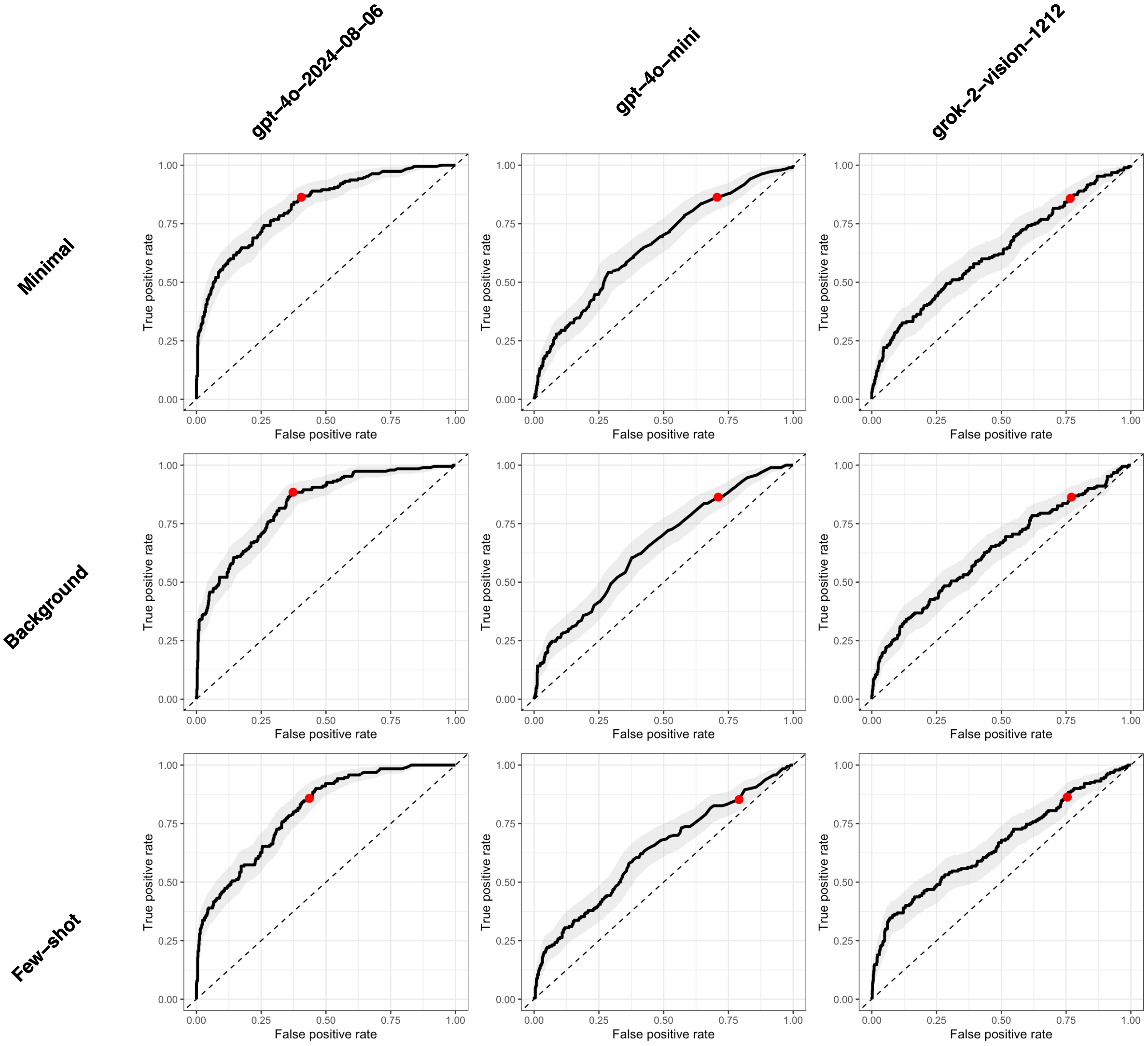

Supplementary Figure 2: ROC curves with 95% confidence intervals (CI) for true positive rate (sensitivity) shown as shaded regions. Set points determined as the sensitivity for which the 95% CI lower bound was ≥ 80% are illustrated with red markers.

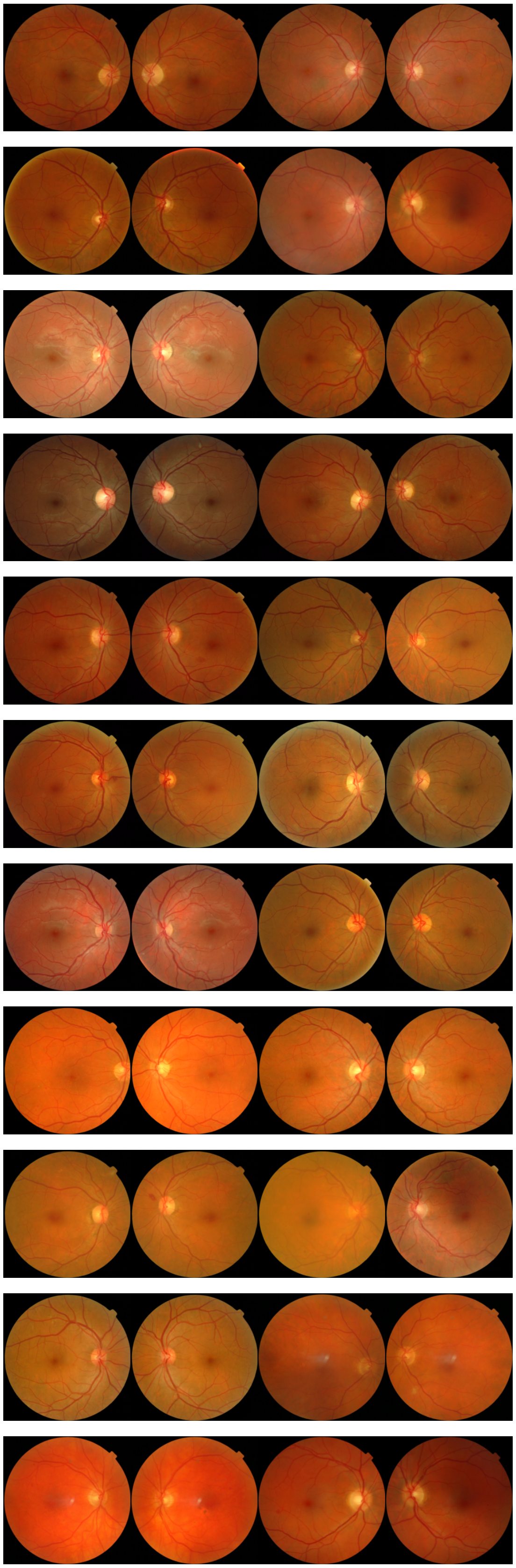

Supplementary Figure 3: All false negative predictions for gpt-4o-2024-08-06 with the background prompting strategy. The resolution shown is the resolution provided to the models, 768 x 768 pixels.
